# *CYP17A1–ATP2B1* SNPs and Gene–Gene and Gene–Environment Interactions on Essential Hypertension

**DOI:** 10.3389/fcvm.2021.720884

**Published:** 2021-10-14

**Authors:** Bi-Liu Wei, Rui-Xing Yin, Chun-Xiao Liu, Guo-Xiong Deng, Yao-Zong Guan, Peng-Fei Zheng

**Affiliations:** ^1^Department of Cardiology, Institute of Cardiovascular Diseases, The First Affiliated Hospital, Guangxi Medical University, Nanning, China; ^2^Guangxi Key Laboratory Base of Precision Medicine in Cardio-Cerebrovascular Disease Control and Prevention, Nanning, China; ^3^Guangxi Clinical Research Center for Cardio-Cerebrovascular Diseases, Nanning, China

**Keywords:** *CYP17A1*, *ATP2B1*, single nucleotide polymorphisms, interactions, hypertension

## Abstract

**Background:** The association between the *CYP17A1* and *ATP2B1* SNPs and essential hypertension (referred to as hypertension) is far from being consistent. In addition to the heterogeneity of hypertension resulting in inconsistent results, gene–gene and gene–environment interactions may play a major role in the pathogenesis of hypertension rather than a single gene or environmental factor.

**Methods:** A case–control study consisting of 1,652 individuals (hypertension, 816; control, 836) was conducted in Maonan ethnic minority of China. Genotyping of the four SNPs was performed by the next-generation sequencing technology.

**Results:** The frequencies of minor alleles and genotypes of four SNPs were different between the two groups (*p* < 0.001). According to genetic dominance model analysis, three (rs1004467, rs11191548, and rs17249754) SNPs and two haplotypes (*CYP17A1* rs1004467G-rs11191548C and *ATP2B1* rs1401982G-rs17249754A) were negatively correlated, whereas rs1401982 SNP and the other two haplotypes (*CYP17A1* rs1004467A-rs11191548T and *ATP2B1* rs1401982A-rs17249754G) were positively associated with hypertension risk (*p* ≤ 0.002 for all). Two best significant two-locus models were screened out by GMDR software involving SNP–environment (rs11191548 and BMI ≥ 24 kg/m^2^) and haplotype–environment (*CYP17A1* rs1004467G-rs11191548C and BMI ≥ 24 kg/m^2^) interactions (*p* ≤ 0.01). The subjects carrying some genotypes increased the hypertension risk.

**Conclusions:** Our outcomes implied that the rs1004467, rs11191548, and rs17249754 SNPs and *CYP17A1* rs1004467G-rs11191548C and *ATP2B1* rs1401982G-rs17249754A haplotypes have protective effects, whereas the rs1401982 SNP and *CYP17A1* rs1004467A-rs11191548T and *ATP2B1* rs1401982A-rs17249754G haplotypes showed adverse effect on the prevalence of hypertension. Several SNP–environment interactions were also detected.

## Introduction

Essential hypertension (referred to as hypertension) is a regular multifactorial disease affecting about one-fourth of adults worldwide (1). Conversely, most of its potential mechanisms are still unknown. It is well-known that environmental factors, including excessive salt intake, tobacco use, physical inactivity, alcohol abuse, overweight, and obesity, increase blood pressure (BP) levels (2), but about half of population BP changes are determined by genetic factors (3, 4).

Genome-wide association studies (GWASs) can screen and analyze hypertension risk genes (5). For instance, two large GWASs (Global BPgen and CHARGE) have identified 14 risk loci that reached genome-wide significant closely related to BP in 2009, including ATPase, Ca^2+^ transporting, plasma membrane 1 gene (*ATP2B1*) and cytochrome P450, family 17, subfamily A, and polypeptide 1 gene (*CYP17A1*) (6, 7). The results about single-nucleotide polymorphism (SNP) of *ATP2B1* and *CYP17A1* were tested and verified soon afterwards in different ethnic groups (8–12). In particular, the reproductions about *ATP2B1* and *CYP17A1* were also conducted in Chinese Han population according to the GWASs (4, 13). However, the evidence that showed the relationship of *ATP2B1* and *CYP17A1* with the hypertension risk from Maonan being one of China's ethnic minorities was still rare.

The *CYP17A1* encodes the P450c17 protein, a member of the cytochrome P450 superfamily of enzymes speeding up plenty of chemical synthesis processes involving steroids, cholesterin, and other blood fats (14). Recently, some articles have reported that the *CYP17A1* is related to hypertension, and one reason for how this gene leads to hypertension may be that genetic factors can influence the distribution of fat in body, and then lipid metabolism disorders can cause BP elevating (15–19). Several hypertension susceptibility genes are also associated with lipid profile and fat distribution (17–19). For instance, Zhang et al. reported that two SNPs (rs11191548 and rs1004467) in the *CYP17A1* locus were correlated with hypercholesterolemia in Han Chinese (19). In addition, in 2012, a Japanese research also found that the *CYP17A1* rs1004467 SNP was associated with the reduction of two types of fat, including visceral and subcutaneous (17). However, Liu et al. had a different opinion regarding the relationship between the *CYP17A1* polymorphism and body mass index (BMI) (4).

*ATP2B1* is attributed to the family of P-type primary ion transport ATPases (10). The associations of two SNPs (rs1401982, a common intronic variant, and rs17249754, a common intergenic variant with the strongest association of the SNPs) in the *ATP2B1* region with both BP and risk of hypertension susceptibility were previously found by GWASs (6, 7, 11) and replicated in the Japanese (8, 9), Korean (10), East Asian (12), and Chinese populations (13). Wang et al. reported that two loci (rs17249754 and rs1401982) were negatively associated with hypertension in a Chinese population (13). However, a Korean genome epidemiology study showed that *ATP2B1* rs17249754 polymorphism may be increased the incident hypertension, when sodium was excessively consumed (20). Tabara et al. also demonstrated that the rs1401982 minor allele may be at higher risk of hypertension in the Japanese (8). The underlying mechanism of *ATP2B1* affecting BP may be that the *ATP2B1* encodes plasma membrane calcium ATPase with an important function in intracellular calcium homeostasis (21, 22). Therefore, some studies have suggested that *ATP2B1* polymorphism may change arterial stiffness by affecting vascular reactivity (13, 23).

The above studies have shown significant association between the *CYP17A1*–*ATP2B1* SNPs and hypertension, but others also showed no association between them. The contradictory results may be related to the following factors (14): (1) ignoring the influence of environment–environment, environment–gene, and gene–gene interactions on BP parameters; (2) some variants found in GWASs may not be functional and have little effect on BP phenotype; (3) variation found in GWASs may have linkage disequilibrium (LD) with some functional variants rather than their own role; (4) the frequency of a high-risk genotype is not alike in different races. For example, in the International 1000 Genomes database (https://www.ncbi.nlm.nih.gov/variation/tools/1000genomes/), the frequency of rs1004467GG genotype in the Chinese Han population was 0.364, which was slightly higher than the genotype frequency of 0.322 in the Japanese population, but both were significantly higher than that (0.104) in the European population. These differences may be caused by evolutionary divergence, or it may be the result of negative selection of rs1004467 risk alleles in European populations. Therefore, we should continue (1) to evaluate the differences in genotypes and allele frequencies in other populations of different ancestry; (2) to screen larger cohorts with clinical BP abnormalities; and (3) to evaluate gene–gene (G × G) and gene–environment (G × E) interactions on BP and hypertension, which are very meaningful and necessary (14).

Maonan is one of the mountain ethnic groups with a small population in China (24). Its living environment, dietary structure, lifestyle, and genetic background are different from the local Han population (25–28). Our previous popular survey found that the prevalence of hypertension in this ethnic group was higher than that in the local Han population (49 vs. 31%, *p* < 0.001) (24). However, up to now, the reason for these differences in BP levels between the two ethnic groups and their risk factors has not been understood. Therefore, the purpose of this research was to test the association of *ATP2B1* (rs1401982 and rs17249754) and *CYP17A1* (rs1004467 and rs11191548) SNPs, and their haplotypes, G × G and G × E interactions, with hypertension in the Maonan population.

## Methods

### SNP Selection

There were five steps for screening four SNPs of *CYP17A1* and *ATP2B1*: (1) SNPs belonging to tagging SNPs were detected by Haploview (Broad Institute or MIT and Harvard, Cambridge, MA, USA, version 4.2). (2) *CYP17A1* (rs1004467 and rs11191548) and *ATP2B1* (rs1401982 and rs17249754) SNPs were then chosen by SHEsis Main (http://analysis.bio-x.cn/myAnalysis.php). (3) The minor allele frequency (MAF) of the SNPs was more than 1%. (4) SNPs may be associated with hypertension according to the previous investigations. (5) SNP-related information was acquired from NCBI dbSNP Build 132 (http://www.ncbi.nlm.nih.gov/SNP/).

### Research Populations

A total of 1,652 Maonan subjects were randomly extracted from previously stratified random samples to conduct a cross-sectional study of hypertensive molecular epidemiology (29). The participants were aged 18–90 years with an average age of 56.6 ± 13.1 years in controls and 56.7 ± 12.3 years in hypertensives. The detailed description of the selection criteria for Maonan participants can be found in two previous studies (24, 30). Besides, all participants were also demonstrated to be Maonan ethnic group by Y chromosome and mitochondrial diversity studies (31). Subjects had complete data on BP and other laboratory parameters and no various related illnesses such as cardiovascular disease, secondary hypertension, and nephropathy. Calculating sample quantity was performed using quanto software (32). All participants had signed informed consent. All the research programs of this project have been approved by the Ethics Committee of the First Affiliated Hospital of Guangxi Medical University (No: Lunshen-2014-KY-Guoji-001; Mar. 7, 2014) (31).

### Epidemiological Survey

International standardization methods were used for the epidemiological survey (24, 33). Trained health professionals collected data such as demographics, medical history, and lifestyle elements by standardized questionnaires. Alcohol and cigarette usage was designated into either one of two groups (yes or no) (34). BMI (kg/m^2^) was calculated as weight/(height^2^). Sitting BP was determined three times after taking a rest at least 5 min using a manual sphygmomanometer, and the average of three readings was used for BP analysis (24).

### Serum Lipid Measurements

Serum cholesterol (TC), triglyceride (TG), high-density lipoprotein cholesterol (HDL-C), and low-density lipoprotein cholesterol (LDL-C) were tested by commercially available enzyme assays (31), and all the tests were carried out by an automatic analyzer in the Clinical Science Experiment Center of the First Affiliated Hospital, Guangxi Medical University (31, 35).

### Genotyping

The genome DNA was isolated from venous blood white cells with phenol-chloroform (36). All DNA samples were saved at −80°C for the next analysis. Genotyping of the four SNPs was achieved by next-generation sequencing techniques [Sangon Biotech (Shanghai) Co; Ltd] (31). The sense and antisense primers used in this study are shown in Supplementary Table 1.

### Diagnostic Criteria

Hypertension was defined as an average systolic blood pressure (SBP) ≥ 140 mmHg and/or diastolic blood pressure (DBP) ≥ 90 mmHg, or using drugs for treating high BP (37). Hyperlipidemia was diagnosed as an average TC > 5.17 mmol/L, and/or TG > 1.70 mmol/L (31, 38). Age subgroup was divided into two groups: <60 and ≥60 years (34, 35). A BMI <24, 24–28, and > 28 kg/m^2^ was defined as normal weight, overweight, and obesity, respectively (36).

### Statistical Analyses

Statistical analyses of the data were realized by the SPSS 22.0 (31), which was the statistical software (SPSS Inc., Chicago, IL, USA). Differences in quantitative data of normal distribution, non-normally distributed data, and qualitative data between hypertension and control participants were analyzed by *t*-test, Wilcoxon–Mann–Whitney test, and chi-square test, respectively. The analyses of Hardy–Weinberg equilibrium (HWE), genotype and allele frequencies, pairwise LD, and haplotype frequencies were mainly performed by the SHEsis online genetics software (http://analysis.bio-x.cn/myAnalysis.php) (31, 39). Logistic regression analyses employed not only the association between SNPs and hypertension, but also the interactions of G × G and G × E on the risk of hypertension after adjustment of sex, age, cigarette smoking, drinking, BMI, and hyperlipidemia (35, 36). A *p*-value < 0.05 was considered statistically significant. The best G × G and G × E interaction combination was screened by Generalized multifactor dimensionality reduction (GMDR) (31, 40–42). Then, the best model with the maximation of cross-validation consistency was chosen (36, 43). Finally, the prediction accuracy of the recognition model was statistically tested by a sign test (providing empirical *p*-values) (31). G × G and G × E interactions of the best model were presented by hierarchical interaction graphs and interaction dendrograms of MDR (43). Besides, traditional statistical approaches were applied to test the outcomes from MDR analyses, and *p* <0.016 was considered statistically significant after Bonferroni correction (0.05/3) (36, 43).

## Results

### Demographic Characteristics

The demographic parameters of 1,652 subjects are shown in Table 1. Compared with the control group, hypertensive patients had higher BMI, SBP, DBP, blood glucose, TC, TG, and LDL-C, but lower HDL-C (*p* < 0.001). However, there was no difference in age, sex ratio, smoking, and drinking between the control and case groups (*p* > 0.05 for all).

**Table 1:** General characteristics of the study subjects.

**Parameter**	**Control**	**Hypertension**	***t*(χ^**2**^)**	***p***
Number	836	816		
Age (years)	56.7 ± 13.1	56.7 ± 12.3	−0.01	0.99
Body mass index (kg/m^2^)	22.9 ± 4.47	25.3 ± 4.14	−10.79	<0.001
Waist circumference (cm)	77.4 ± 9.6	83.3 ± 10.2	−11.96	<0.001
Systolic blood pressure (mmHg)	119 ± 11	151 ± 16	−46.34	<0.001
Diastolic blood pressure (mmHg)	74 ± 8	92 ± 10	−37.88	<0.001
Glucose (mmol/L)	6.06 ± 1.32	6.43 ± 1.46	−5.41	<0.001
Total cholesterol (mmol/L)	4.87 ± 0.88	5.18 ± 0.99	−6.74	<0.001
Triglyceride (mmol/L)	1.26 (0.93)	1.68 (1.18)	−10.55	<0.001
HDL-C (mmol/L)	1.30 ± 0.23	1.24 ± 0.31	4.45	<0.001
LDL-C (mmol/L)	3.08 ± 0.41	3.25 ± 0.55	−7.28	<0.001
Male/female	426/410	415/401	0.002	0.97
**Smoking status [*****n*** **(%)]**				
Non-smoker	614 (73.4)	608 (74.5)		
Smoker	222 (26.6)	208 (25.5)	0.24	0.62
**Alcohol consumption [*****n*** **(%)]**				
Non-drinker	659 (78.8)	633 (77.6)		
Drinker	177 (21.2)	183 (22.4)	0.38	0.54

*Normal distribution quantitative data are presented as mean ± SD. Non-normal distribution data such as triglyceride are expressed as median (interquartile range). Qualitative variables are expressed as percentages (%). LDL-C, low-density lipoprotein cholesterol; HDL-C, high-density lipoprotein cholesterol*.

### Genotype and Allele Frequencies and Hypertension

**Figure 1:**
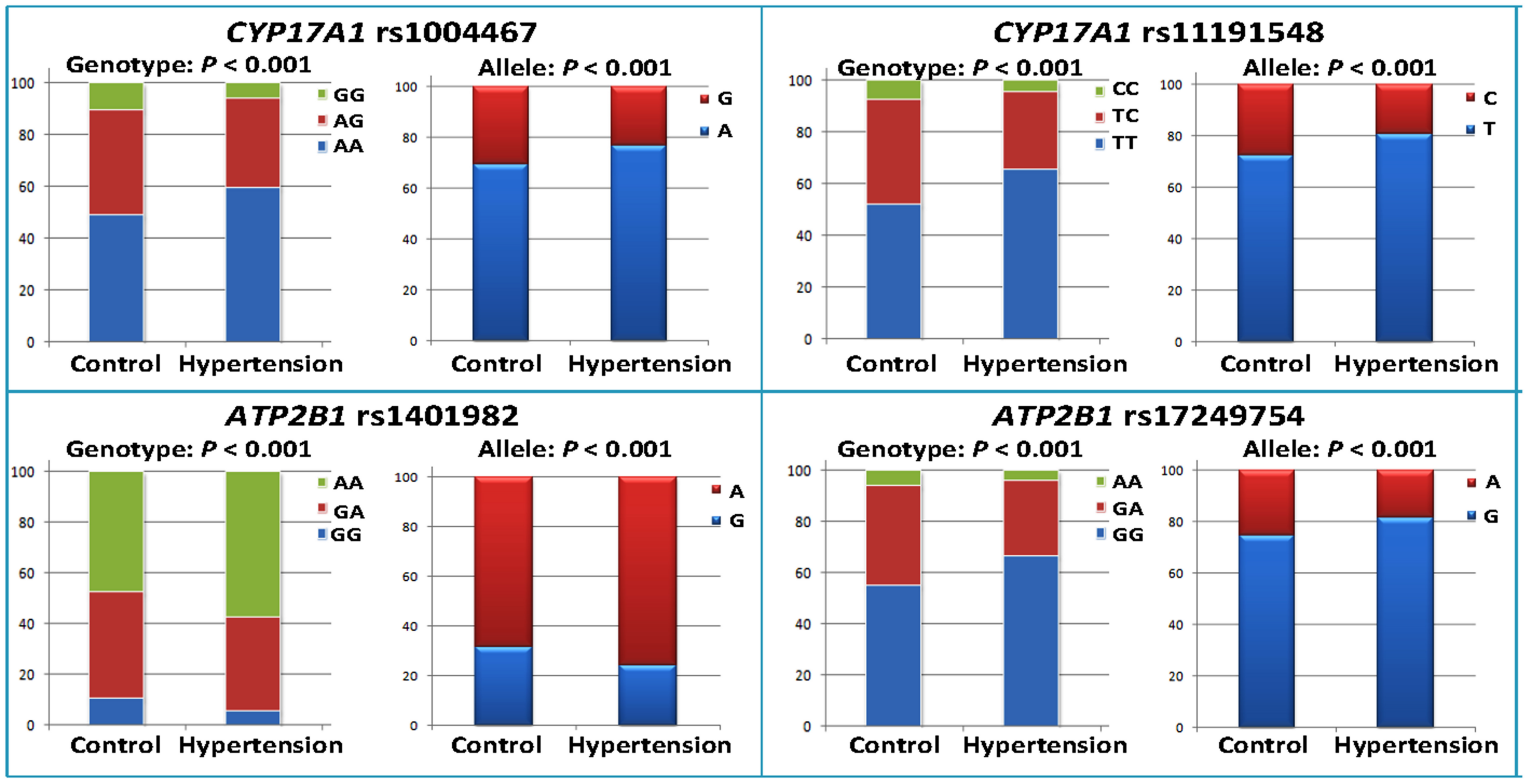
Genotype and allele frequencies of the four *CYP17A1*–*ATP2B1* SNPs in the control and hypertension groups.

As shown in Table 2, the minor allele and genotype distribution of the rs1004467, rs11191548, rs1401982, and rs17249754 SNPs was different between the patient and control groups (*p* < 0.001). Figure 1 shows the genotype and allele frequencies of each SNP in control and hypertension groups. The genotype distribution was consistent with the HWE (*p* > 0.05 for all). Simultaneously, the rs1401982 SNP enhanced the risk of hypertension, whereas the rs1004467, rs11191548, and rs17249754 SNPs decreased the susceptibility of hypertension in the dominant model (*p* ≤ 0.002 for all).

**Table 2:** Correlation between the *CYP17A1*–*ATP2B1* polymorphisms and hypertension.

**SNP**	**Genotype**	**Control** **(*n* = 836)**	**Hypertension** **(*n* = 816)**	**χ^**2**^**	***p***	**Adjusted OR** **(95% CI)**	*p
***CYP17A1***	AA	410 (49.0)	487 (59.7)	22.9	1.07E-005	1	–
**rs1004467 A>G**	AG+ GG	426 (51.0)	329 (40.3)			0.66 (0.54–0.81)	<0.001
	MAF	511 (30.6)	376 (23.0)	23.8	1.09E-006		
	*P*_HWE_	0.26	0.47				
***CYP17A1***	TT	436 (52.1)	538 (65.9)	33.08	6.55E-008	1	–
**rs11191548T>C**	TC+ CC	400 (47.9)	278 (34.1)			0.57 (0.46–0.7)	<0.001
	MAF	461 (27.6)	314 (19.2)	31.93	1.64E-008		
	*P*_HWE_	0.66	0.19				
***ATP2B1***	GG	91 (10.9)	47 (5.8)	22.42	1.36E-005	1	–
**rs1401982 G>A**	GA+ AA	745 (89.1)	769 (94.2)			1.83 (1.24–2.7)	0.002
	MAF	1142 (68.3)	1235 (75.7)	22.24	2.45E-006		
	*P*_HWE_	0.26	0.81				
***ATP2B1***	GG	460 (55.0)	544 (66.7)	24.06	5.97E-006	1	
**rs17249754 G>A**	GA+ AA	376 (45.0)	272 (33.3)			0.68 (0.55–0.84)	<0.001
	MAF	425 (25.4)	302 (18.5)	23.0	1.65E-006		
	*P*_HWE_	0.36	0.63				

CYP17A1, cytochrome P450 17A1; ATP2B1, ATPase, Ca^2+^ transporting, plasma membrane 1; MAF, minor allele frequency; HWE, Hardy–Weinberg equilibrium. p is the probability of chi-square test; *p is the probability of logistic regression analyses. The symbol “–” means there is no data.

### Haplotypes and the Risk of Hypertension

LD analysis showed that the four SNPs did not have statistical independence in the control or case group. However, the LD between the rs1004467 and rs11191548 (*D*' = 0.950) or between the rs1401982 and rs17249754 SNPs (*D*' = 0.951) was strong in both control and hypertension groups (Supplementary Figure 1; Supplementary Table 2). As shown in Table 3, the most common haplotypes were *CYP17A1* rs1004467A-rs11191548T and *ATP2B1* rs1401982A-rs17249754G (≥ 67% of the samples). The frequencies of *CYP17A1* rs1004467A-rs11191548T, *CYP17A1* rs1004467G-rs11191548C, *ATP2B1* rs1401982A-rs17249754G, and *ATP2B1* rs1401982G-rs17249754A haplotypes were significantly different between the control and case groups. Meanwhile, the haplotypes of *CYP17A1* rs1004467A-rs11191548T, *CYP17A1* rs1004467G-rs11191548C, and *ATP2B1* rs1401982G-rs17249754A had a protective effect for hypertension, whereas the haplotype of *ATP2B1* rs1401982A-rs17249754G revealed an increased susceptibility of disease (*p* < 0.001).

**Table 3:** Association between the haplotypes and hypertension risk.

**Haplotype**	**Hypertension Fre**.	**Control Fre**.	**χ^**2**^**	***p***	**OR (95% CI)**
rs1004467A-rs11191548C	12.56 (0.008)	15.61 (0.009)	–	–	–
rs1004467A-rs11191548T	1,243.44 (0.76)	1,145.39 (0.69)	24.16	9.04E-007	1.48 (1.26–1.72)
rs1004467G-rs11191548C	301.44 (0.19)	445.39 (0.27)	31.86	1.70E-008	0.62 (0.53–0.73)
rs1004467G-rs11191548T	74.56 (0.05)	65.61(0.04)	0.83	0.36	1.17 (0.83–1.64)
rs1401982 A-rs17249754A	9.61 (0.006)	16.16 (0.010)	–	–	–
rs1401982A-rs17249754G	1,225.39 (0.75)	1,125.84 (0.67)	22.96	1.68E-006	1.45 (1.25–1694)
rs1401982G-rs17249754A	292.39 (0.18)	408.84 (0.25)	21.67	3.28E-006	0.67 (0.57–0.79)
rs1401982G-rs17249754G	104.61 (0.06)	121.16 (0.07)	0.97	0.33	0.87 (0.67–1.15)

*The haplotype is combined with CYP17A1 rs1004467-rs11191548 and ATP2B1 rs1401982-rs17249754. Control Fre., the frequency of haplotypes in control individuals; Hypertension Fre., the frequency of haplotypes in hypertension subjects. Rare Hap (frequency <3%) has been ignored in control and case subjects*.

### G × G and G × E Interaction on Hypertension

The GMDR model was utilized to analyze the interaction of G × G and G × E among SNPs, haplotypes, BMI, age, gender, alcohol, and/or cigarette usage on the risk of hypertension. Table 4 summarizes the results of G × G and G × E interactions of the two and three loci models derived from GMDR analysis. A significant two-locus model revealed a potential SNP–environment interaction between the rs11191548 SNP and BMI ≥ 24 kg/m^2^ (*p* = 0.01), with a cross-validation consistency (7/10) and a testing accuracy of 62.7%. Another significant two-locus model (*CYP17A1* rs1004467G-rs11191548C and BMI ≥ 24 kg/m^2^, *p* = 0.0004) indicated a potential haplotype–environment interaction, with a cross-validation consistency (9/10) and a testing accuracy of 63.5%.

**Table 4:** GMDR analysis of SNPs, haplotypes, and environments showed different interactions.

**Locus no**.	**Best combination**	**Training Bal. Acc**	**Testing Bal. Acc**	**Cross-validation consistency**	**χ^**2**^**	***p***	**OR (95% CI)**
**SNP–SNP interaction**							
**2**	Rs11191548, rs17249754	0.58	0.56	8/10	1.55	0.21	1.71 (0.69, 4.2)
**3**	Rs1004467, rs11191548, rs1401982	0.59	0.57	8/10	1.82	0.18	1.82 (0.74, 4.47)
**SNP–environment interaction**							
**2**	Rs11191548, BMI ≥ 24	0.64	0.63	7/10	6.07	0.01	3.26 (1.27, 8.38)
**3**	Rs11191548, BMI ≥ 24, gender	0.66	0.64	6/10	6.81	0.009	3.41 (1.35, 8.61)
**Haplotype–haplotype interaction**							
**2**	G-C, A-G	0.56	0.56	9/10	2.58	0.11	1.64 (0.88, 3.06)
**3**	A-T, G-T, A-G	0.56	0.56	10/10	3.11	0.08	1.72 (0.93, 3.21)
**Haplotype–environment interaction**							
**2**	G-C, BMI ≥ 24	0.64	0.63	9/10	12.75	0.0004	3.23 (1.69, 6.18)
**3**	Age, BMI ≥ 24, G-C	0.64	0.64	7/10	12.51	0.0004	3.12 (1.65, 5.9)

*p, adjusting for gender, age, smoking, alcohol consumption, BMI, and hyperlipidemia. The haplotype is combined with CYP17A1 rs1004467-rs11191548 and ATP2B1 rs1401982-rs17249754*.

**Figure 2:**
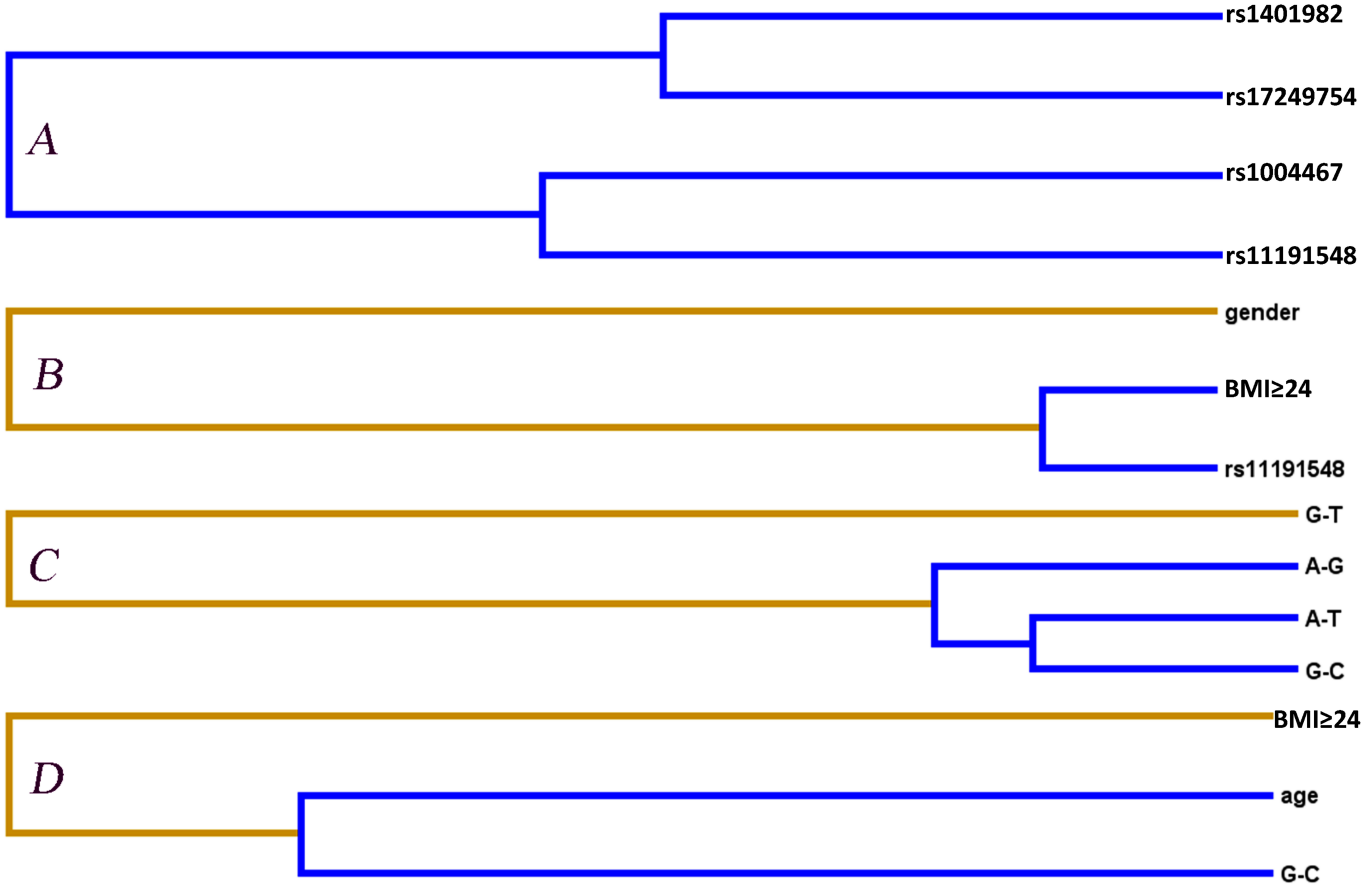
Different types of interaction dendrogram for gene–gene **(A,C)** and gene–environment **(B,D)** on the hypertension risk. Orange color (synergy); blue color (strong antagonism interaction). The elements with stronger interaction are closer at the leaves of the tree.

Entropy-based interaction dendrograms obtained from MDR analysis are shown in Figure 2, which exhibited the strongest antagonistic effect of the SNP–SNP interaction (rs1401982 and rs17249754), SNP–environment interaction (rs11191548 and BMI ≥ 24 kg/m^2^), haplotype–haplotype interaction (*CYP17A1* rs1004467A-rs11191548T and *CYP17A1* rs1004467G-rs11191548C), and haplotype–environment interaction (*CYP17A1* rs1004467G-rs11191548C and age), respectively. In order to obtain the OR and 95% CI for the joint effects, we implemented an interaction study by logistic regression analyses (Table 5). When the SNP–environment interaction was analyzed, we found that the individuals with rs11191548 TC/CC genotypes and BMI ≥ 24 kg/m^2^ raised the risk of hypertension (adjusted OR = 1.45, 95% CI = 1.08–1.94, *p* = 0.014) compared to the individuals with rs11191548 TT and BMI ≥ 24 kg/m^2^.

**Table 5:** Various types of interactions were analyzed by logistic regression analysis.

**Variable 1**	**Variable 2**	**OR (95% CI)**	***P***
**SNP–SNP interaction**			
Rs1401982	Rs17249754		
GG	No	1	–
GG	Yes	1.55 (0.24–10.27)	0.65
GA+AA	No	3.12 (0.49–19.88)	0.23
GA+AA	Yes	2.26 (0.35–14.49)	0.39
**SNP–environment interaction**			
Rs11191548	BMI ≥ 24		
TT	No	1	
TT	Yes	4.38 (3.31–5.79)	<0.001
TC+CC	No	1.03 (0.76–1.39)	0.87
TC+CC	Yes	1.45 (1.08–1.94)	0.014
**Haplotype–haplotype interaction**			
A-T	G-C		
Non-carriers	Non-carriers	1	
Non-carriers	Carriers	0.63 (0.44–0.91)	0.012
Carriers	Non-carriers	1.02 (0.73–1.42)	0.92
Carriers	Carriers	–	–
**Haplotype–environment interaction**			
G-C	Age ≥ 60		
Non-carriers	No	1	–
Non-carriers	Yes	1.61 (1.35–1.91)	<0.001
Carriers	No	0.78 (0.62–0.99)	0.041
Carriers	Yes	0.75 (0.58–0.96)	0.023

*P, adjusting for gender, age, smoking, alcohol consumption, BMI, and hyperlipidemia. G-C and A-T are combined with CYP17A1 rs1004467-rs11191548. p < 0.016 was considered statistically significant after Bonferroni correction (0.05/3)*.

## Discussion

In this cross-sectional study of hypertensive molecular epidemiology, the association of the *ATP2B1* and *CYP17A1* SNPs, and their haplotypes, G × G and G × E interactions, with hypertension in the Maonan population was observed for the first time. The main findings are as follows: (1) The genotype and allele frequencies of the *CYP17A1* rs1004467, *CYP17A1* rs11191548, *ATP2B1* rs1401982, and *ATP2B1* rs17249754 SNPs were significantly different between the control and hypertension groups. (2) The *ATP2B1* rs1401982 SNP enhanced the risk of hypertension, whereas the *CYP17A1* rs1004467, *CYP17A1* rs11191548, and *ATP2B1* rs17249754 SNPs decreased the prevalence of hypertension in the dominant models. (3) The frequencies of *CYP17A1* rs1004467A-rs11191548T, *CYP17A1* rs1004467G-rs11191548C, *ATP2B1* rs1401982A-rs17249754G, and *ATP2B1* rs1401982G-rs17249754A haplotypes were significantly different between the control and case groups. (4) The *CYP17A1* rs1004467A-rs11191548T, *CYP17A1* rs1004467G-rs11191548C, and *ATP2B1* rs1401982G-rs17249754A haplotypes had a protective effect for hypertension, whereas the *ATP2B1* rs1401982A-rs17249754G haplotype increased the risk of hypertension. (5) Several interactions including rs11191548-BMI ≥ 24 kg/m^2^ (SNP–environment) and rs1004467G-rs11191548C-BMI ≥ 24 kg/m^2^ (haplotype–environment) on the risk of hypertension were also observed. (6) The individuals with rs11191548 TC/CC genotypes and BMI ≥ 24 kg/m^2^ raised the risk of hypertension.

In the past 10 years, according to the results of GWAS scans, both *ATP2B1* and *CYP17A1* have correlation with BP and/or hypertension (6, 7). However, the genetic association between the *ATP2B1* or *CYP17A1* and hypertension was conflicting. The most important reasons for the discrepant outcomes may be that hypertension is a complicated illness that is influenced by various environmental elements, small effect polygenes, and their interactions (44). The genotype and allele frequencies of the *CYP17A1*–*ATP2B1* SNPs are different in distinct races, ethnic groups, or populations according to the International 1,000 Genomes database (https://www.ncbi.nlm.nih.gov/variation/tools/1000genomes/). The *CYP17A1* rs1004467G allele frequency in Chinese Dai in Xishuangbanna, China (CDX), Han Chinese in Beijing, China (CHB), and Southern Han Chinese (CHS) was 28.49, 36.41, and 35.71%, respectively. The *CYP17A1* rs11191548C allele frequency in CDX, CHB, and CHS was 25.27, 29.61, and 28.10%, respectively. The *ATP2B1* rs1401982G allele frequency in CDX, CHB, and CHS was 27.96, 34.95, and 39.05%, respectively. The *ATP2B1* rs17249754A allele frequency in CDX, CHB, and CHS was 18.82, 32.04, and 36.67%, respectively. In the present study, we found that the MAF of these SNPs was lower than other Chinese, especially in the hypertension group, but it was higher in our study populations than in Europeans or Africans. These results might also be a reasonable explanation for the distinct prevalence of hypertension between Chinese and Europeans or Africans.

Maonan people not only like to eat beef, pork, and animal viscera, all of which are rich in saturated fatty acid, but also like sour marinated meat, snails, and sour pickles that contain a lot of salt (36). High-fat diet is an important element leading to obesity, dyslipidemia (45), atherosclerosis, and hypertension (46, 47). In particular, high-salt diet has a significant impact on hypertension (2, 4, 15). Therefore, the eating habits of Maonan residents may explain the differences in BMI, BP, TC, and TG values between the control and case groups.

There was no statistical significance in alcohol and cigarette consumption rates between control and hypertension groups in our research. The effects of drinking and smoking on hypertension have been reported by previous articles. The extent to which alcohol is associated with hypertension may be partly related to the amount of alcohol consumed (48–51). Low levels of alcohol use mean no different from or slightly lower BP (48–51), and high levels of alcohol consumption are a strong predictor of the high BP risk (48, 52). Smokers usually have higher BP than non-smokers (53, 54). However, the effects of alcohol and tobacco on the risk of hypertension in many studies were still inconsistent (48–51). We assume that these discrepancies could be due to numerous factors, including sample size, misclassification bias according to participants' self-reported questionnaires, ethnicities, age groups, and gender, warranting that further research should take into account the factors above (48–51, 55, 56). To address the possibility that many genetic variants associated with hypertension found by the GWASs might be the result of different environmental as well as direct genetic effects (57), our study used some examples, including eating habits and alcohol and cigarette consumption.

In the current study, minor allele and genotype frequencies of all four SNPs had a difference in control and case groups (*p* < 0.001). These results showed that *CYP17A1* and *ATP2B1* SNPs were correlated with hypertension and genetic factors might play a part in susceptibility to hypertension. Furthermore, according to genetic dominance model analysis, three SNPs (rs1004467, rs11191548, and rs17249754) and two haplotypes (*CYP17A1* rs1004467G-rs11191548C and *ATP2B1* rs1401982G-rs17249754A) were negatively correlated with hypertension risk, while the rs1401982 SNP and the other two haplotypes (*CYP17A1* rs1004467A-rs11191548T and *ATP2B1* rs1401982A-rs17249754G) were positively associated with hypertension risk (*p* ≤ 0.002). Meanwhile, GMDR analysis showed no statistical difference between the interaction of *CYP17A1* and *ATP2B1* on hypertension. However, two best significant two-locus models were screened out involving SNP–environment (rs11191548 and BMI ≥ 24 kg/m^2^) and haplotype–environment (*CYP17A1* rs1004467G-rs11191548C and BMI ≥ 24 kg/m^2^) interactions (*p* ≤ 0.01). The participants with the rs11191548 TT genotype and BMI ≥ 24 kg/m^2^ had higher risk of hypertension than the individuals with the rs11191548 TC/CC genotypes and BMI ≥ 24 kg/m^2^. G × E interaction on the development of hypertension was also observed in this cross-sectional study.

The prevalence of hypertension is increasing year by year, so new and more effective measures are urgently needed to prevent and treat hypertension. However, this depends on the discovery of mechanism of BP regulation. Although lifestyle intervention can successfully reduce BP in some patients, there are still a number of patients with hypertension who need new drugs to decrease BP. GWASs have confirmed that the *ATP2B1* encoding plasma membrane Ca^2+^ ATPase 1 (PMCA1) is strongly associated with BP and hypertension. Several studies have confirmed that PMCA1 plays a physiological role in regulating BP and resistance artery function. PMCA1 may be a potential target for the treatment of essential hypertension (58). At present, the specific mechanism of hypertension has not been fully clarified, and further studies are needed to explore this. This study may provide new information and ideas for the scientists in this field.

There are several potential limitations in our study. First, the number of controls and patients with hypertension was relatively small. Larger samples are necessary to confirm our findings in this study. Second, the general characteristics of the two study populations were different. The potential effects of these factors on BP and hypertension could not be completely eliminated even if the statistical analyses were adjusted. Third, a small number of patients with hypertension received some secondary prevention drugs. Some of these drugs may have a certain effect on BP and hypertension. Fourth, it is worth noting that the four SNPs tested in this study may have LD with some functional variants rather than their own role on BP and hypertension. Fifth, diet and physical activity have a significant impact on BP and hypertension. The statistical analysis of this study failed to adjust the effects of dietary nutrients and physical activity intensity on BP and hypertension. This is also the deficiency of this article. Finally, statistical significance is not entirely consistent with biological significance.

## Conclusions

Our outcomes implied that the rs1004467, rs11191548, and rs17249754 SNPs and *CYP17A1* rs1004467G-rs11191548C and *ATP2B1* rs1401982G-rs17249754A haplotypes revealed protective effects on hypertension, whereas the rs1401982 SNP and *CYP17A1* rs1004467A-rs11191548T and *ATP2B1* rs1401982A-rs17249754G haplotypes showed adverse effect on the prevalence of hypertension. The rs11191548-BMI ≥ 24 kg/m^2^ interaction on hypertension was also observed.

## Data Availability Statement

The data presented in the study are deposited in the Supplementary Material.

## Ethics Statement

The studies involving human participants were reviewed and approved by the Ethics Committee of the First Affiliated Hospital of Guangxi Medical University (No. Lunshen-2014 KY-Guoji-001, Mar. 7, 2014). The patients/participants provided their written informed consent to participate in this study.

## Author Contributions

B-LW conceived the research, took part in design, performed genotyping and statistical analysis, and drafted the manuscripts. R-XY conceived the research, took part in the design, conducted the epidemiological investigation, collected the samples, and helped to draft the manuscript. C-XL collaborated to the genotyping. G-XD, Y-ZG, and P-FZ conducted the epidemiological investigation and helped to collect the samples. All authors contributed to the article and approved the submitted version.

## Funding

This work was supported by the National Natural Science Foundation of China (No. 81460169). There was no role of the funding body in the design of the study and collection, analysis, and interpretation of data and in writing the manuscript.

## Conflict of Interest

The authors declare that the research was conducted in the absence of any commercial or financial relationships that could be construed as a potential conflict of interest.

## Publisher's Note

All claims expressed in this article are solely those of the authors and do not necessarily represent those of their affiliated organizations, or those of the publisher, the editors and the reviewers. Any product that may be evaluated in this article, or claim that may be made by its manufacturer, is not guaranteed or endorsed by the publisher.
