# Supplementary material for: CYP17A1–ATP2B1 SNPs and Gene–Gene and Gene–Environment Interactions on Essential Hypertension
Source: Front Cardiovasc Med. 2021 Oct 14;8:720884. doi: 10.3389/fcvm.2021.720884 (PMC8552967; doi:10.3389/fcvm.2021.720884)
Supplement: Supplementary file 1 [file Data_Sheet_1.docx]

Supplementary Materials

# Supplementary Table 1 | Sense primers and antisense primers

| SNP | Sense primers | Antisense primers |
| --- | --- | --- |
| rs1004467 | 5′-GTCATTATCTGGAGTACTAAGGTGCATAA-3′ | 5′-GGCATCATAGACAACCTGAGCAAA-3′ |
| rs11191548 | 5′-CTGGATTCCCTGATCACCACTAA-3′ | 5′-AAGGCTGCCTGGTCATTTTAAAATAAATAC-3′ |
| rs1401982 | 5′-CATATTAGGGCAGAAACACAGTGAT-3′ | 5′-GTAGTTGTTGCTTTATCAAGGTTTGAGATG-3′ |
| rs17249754 | 5′-GCGACTCCAGTTTTGCTCTTAT-3′ | 5′-GCAAGGTCTTGGAGCAGACTTG-3′ |

**Supplementary Table 2** | Linkage disequilibrium analysis of the *CYP17A1-ATP2B1* SNPs in the hypertension and control groups

| *r*^2^ | rs11191548 | rs1401982 | rs17249754 |
| --- | --- | --- | --- |
| rs1004467 | 0.754 | 0.003 | 0.007 |
| rs11191548 | - | 0.012 | 0.021 |
| rs1401982 | - | - | 0.654 |

The symbol “–” means there is no data.

**
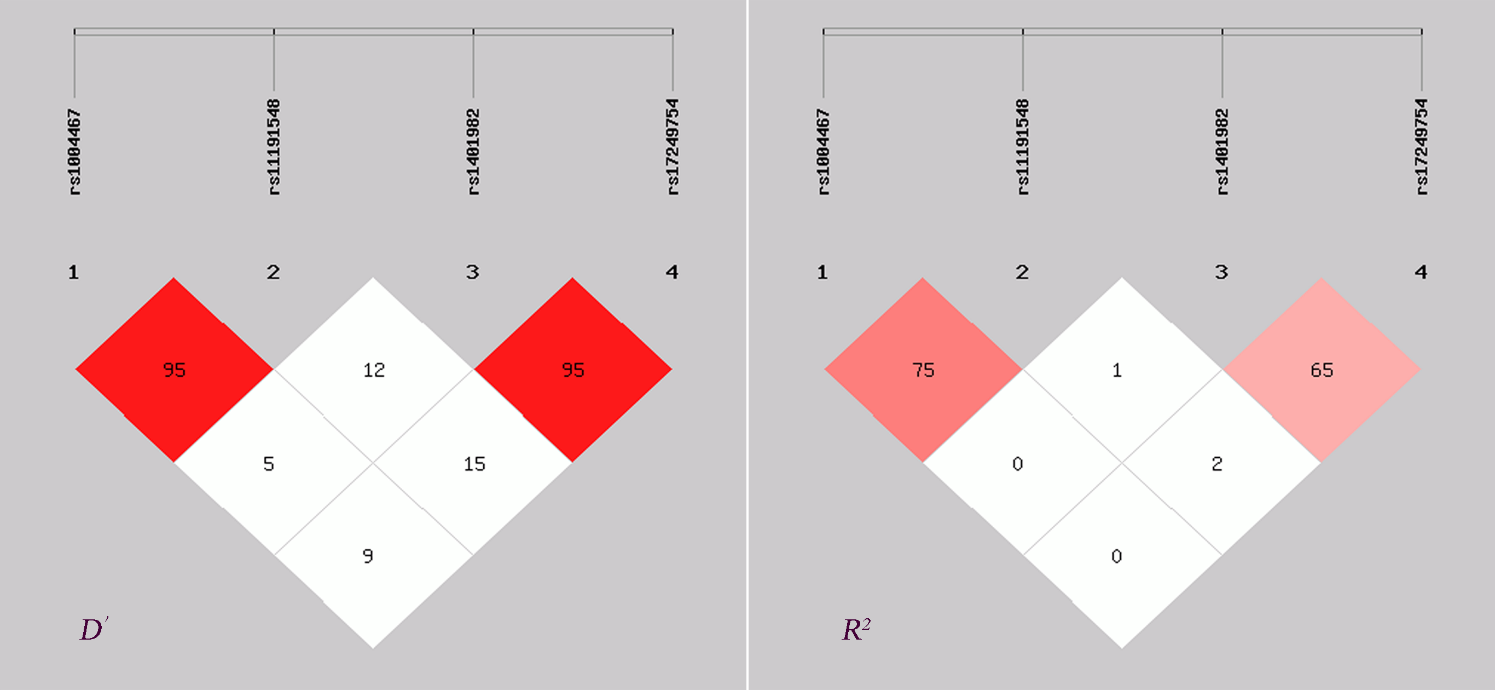
**

**Supplementary Figure 1** | Pair-wise *D'* and *R*^2^ represent the linkage disequilibrium (LD) of *CYP17A1* and *ATP2B1* SNPs in both groups, respectively. The greater the *D'* and *R^2^* values and the darker the color, the higher the LD.
